# Fungal Biotransformation of Hazardous Organic Compounds in Wood Waste

**DOI:** 10.3390/molecules28124823

**Published:** 2023-06-17

**Authors:** Magdalena Komorowicz, Dominika Janiszewska-Latterini, Anna Przybylska-Balcerek, Kinga Stuper-Szablewska

**Affiliations:** 1 Łukasiewicz Research Network—Poznan Institute of Technology, 60-654 Poznan, Poland; dominika.janiszewska@pit.lukasiewicz.gov.pl; 2 Faculty of Forestry and Wood Technology, Department of Chemistry, Poznan University of Life Sciences, 60-628 Poznan, Poland; anna.przybylska@up.poznan.pl (A.P.-B.); kinga.stuper@up.poznan.pl (K.S.-S.)

**Keywords:** wood waste biodegradation, white-rot fungi, PCP biotransformation, lindane biotransformation, PAH biotransformation, biotransformation pathways, metabolites

## Abstract

A diverse spectrum of organisms, such as fungi, bacteria, and actinomycetes, can degrade and transform organic matter, including wood, into valuable nutrients. A sustainable economy has the goal of efficiently using waste as raw materials, and in this optic, it uses biological preparations more and more often, supporting the decomposition of lignocellulosic waste. With reference to wood wastes, which are produced in a substantial amount by the forest and wood industry, one of the possibilities to biodegrade such lignocellulosic material is the composting process. In particular, microbiological inoculum containing dedicated fungi can contribute to the biodegradation of wood waste, as well as the biotransformation of substances from the protection of wood, such as pentachlorophenol (PCP), lindane (hexachlorobenzene) and polycyclic aromatic hydrocarbons (PAHs). The purpose of this research was to produce a literature review in terms of the selection of decay fungi that could potentially be used in toxic biotransformation unions. The findings of the literature review highlighted how fungi such as *Bjerkandera adusta*, *Phanerochaete chrysosporium*, and *Trametes versicolor* might be ingredients of biological consortia that can be effectively applied in composting wood waste containing substances such as pentachlorophenol, lindane, and polycyclic aromatic hydrocarbons (PAHs).

## 1. Introduction

Wood is a natural biopolymer made up of cellulose, hemicelluloses, and lignin. It also contains many secondary substances, e.g., inorganics, terpenes, esters, fatty acids, phenolics, proteins, and alkaloids [1,2,3,4], which perform specific functions during the life of trees.

Wood, as a material of natural origin, is subject to the action of many factors causing mechanical damage or biological decomposition. Structural compounds of wood, as a result of the action of organisms such as fungi and bacteria, are degraded, and as a consequence, this leads to a reduction in the properties of wood and a shortening of its durability.

Due to the fact that wood is a valuable building and construction material, it is important to extend its durability, especially considering that the majority of wood waste is nowadays directly disposed of. Wooden objects, buildings, or architectural elements are protected against deterioration of their physical and mechanical properties by using preservatives. Apart from fungicides and insecticides, such impregnations also include resins, pigments, and other chemicals. Despite the recent efforts to reduce the number of chemicals in wood preservatives, some substances cannot be completely eliminated.

The most famous substances used to protect wood are pentachlorophenol (PCP), lindane (γ-hexachlorophenol; hexachlorocyclohexane), and creosote containing, among others, polycyclic aromatic hydrocarbons (PAHs). These substances were previously used to protect against biological degradation caused by fungi, insects, and other pests (termites, fungi, mites, and other pests) [5]. Currently, due to their toxic effects on the environment and humans, the use of these substances in many countries has been prohibited or permitted only to a limited extent [5,6].

These substances are hazardous because they can be found in wood waste sent to landfills or recycled wood waste. Indeed, notwithstanding the wide interest in using wood as renewable energy feedstock [7,8,9], high risk to the environment is also associated with the energetic use of wood waste [10] as a consequence of the harmful emissions related to the presence of hazardous compounds. In the current situation of wood deficiency and following the statements of the circular economy approach, it should be a natural step to reuse wood waste as much as possible [11,12].

The use of wood waste from end-of-life products, building elements, or structural elements causes many technological and ecological difficulties. The diversity of the wood waste stream, the wide spectrum of the substances and finishing materials used, and the potential presence of hazardous compounds necessitates optimal disposal methods. The most commonly used wood for reuse purposes is wood waste meeting high-quality requirements, such as untreated wood or wood containing no hazardous substances.

Other wood waste containing harmful substances derived from wood protection products, including PCP, lindane, and PAHs, are not applicable and constitute an unnecessary ballast for the environment, considering that their improper disposal may create concerns for the environment and human health. One of the many management methods is composting wood waste. It should take place under controlled technological conditions and with the use of agents improving the bioremediation of hazardous substances. In the literature, the use of bacteria as microbiological agents decomposing lignocellulosic waste with the simultaneous biotransformation of hazardous substances has been widely discussed [13]. Numerous studies inform about the beneficial results obtained with the use of bacteria and even fungi for the biodegradation of hazardous substances such as PCP, lindane, and PAHs in liquid and solid media or in artificially contaminated soil samples. Dedicated microorganisms effectively degrade toxic substances into compounds with lower toxicity to the environment or even into substances included in the carbon cycle in nature (Tricarboxylic Acid Cycle—TCA cycle). Aerobic and anaerobic organisms can degrade substances in a variety of conditions and at different concentrations [13,14,15].

In the literature, the topic of bioremediation of wood waste containing hazardous substances derived from wood preservatives has not been often discussed. Few reports describe only the possibility of using fungi in bioremediation processes. Therefore, a literature review has been produced to determine the possibility of using fungi as inoculum components supporting the composting of wood waste containing hazardous substances derived from wood preservatives (PCP, lindane, PAHs).

The aim of this study was to analyze the literature in order to develop a hypothetical pathway of PCP, lindane, and PAHs with the use of inoculum-containing fungi, taking into account the emerging non-toxic metabolites, metabolites with less environmental harmfulness or metabolites involved in the metabolic cycle.

## 2. Characteristics of Selected Components of Wood Protection

Wood, as a material of natural origin, is susceptible to many external biotic and abiotic factors that cause its decomposition or damage to its structure and, as a consequence, a reduction in the strength and durability of the wood. The use of protective coatings containing various chemical agents may slow down the process of biological decomposition of wood. The use of chemical substances guarantees a longer period of use of wooden elements. Unfortunately, it causes an irreversible change in the chemical composition of the wood. The presence of substances that protect the wood can be toxic to organisms present on the surface or inside the wood, but unfortunately, also to organisms in the immediate vicinity. Some of the most dangerous substances used to protect wooden elements are organic compounds: pentachlorophenol, lindane, and creosote oil. The recognized toxic effect of these compounds on the environment, animals, and humans is the reason for withdrawing from the use of these compounds and restrictions in many countries.

Handling hazardous substances during their production and use in wood preservation stages requires special care. Environmental contamination must be prevented, and the risk of exposure of workers must be kept to a minimum. The treatment of wooden elements and their assembly are subject to supervision; moreover, after the expiry of the time of use, the removal of these wooden elements requires appropriate disposal methods to minimize the risk of environmental contamination. It should be borne in mind that harmful substances can be released during processing, dismantling, and storage through emissions and leaching. The uncontrolled use of wood containing harmful substances can affect human health. Wooden elements containing toxic substances should be disposed of in a controlled manner.

### 2.1. Pentachlorophenol

Pentachlorophenol (PCP) (2,3,4,5,6-pentachlorophenol) (Table 1) has been produced since 1830 as an agent protecting wooden elements against decomposition. It was intended to serve as a bactericide, fungicide, herbicide, algicide, insecticide, and molluscicide agent [16]. The properties of PCP have been applied to the wood preserving and treatment industry, particularly for utility poles, fences, and railway ties [16]. PCP is used as a dip treatment in freshly sawn lumber to prevent sap-staining and blue-staining by fungi [16]. PCP is an inhibitor of mitochondrial oxidative phosphorylation, which negatively affects all living organisms. Under its influence, the chemiosmotic gradient created by electron transport is disturbed in cells, which is necessary for the phosphorylation of Adenosine diphosphate (ADP). As a result, changes in cellular metabolism are intensified. PCP is highly toxic to organisms inhabiting the aquatic environment but not very toxic to birds and farm animals. In turn, its presence in water or soil leads to the inhibition of vegetation and plant death [17].

For humans, pentachlorophenol is extremely toxic if ingested or inhaled. Acute inhalation toxicity manifests itself, inter alia, in nasal narrowing, hoarseness, lacrimation, skin redness or rash, and corneal damage. PCP in the form of dust in a concentration over 0.3 mg/m^3^ causes pain, watery eyes, and a cough. At a concentration of 1 mg/m^3^, dyspnea and respiratory failure occur. Contamination of the skin with dust and a solution (2%) causes redness, pain, and rapidly increasing symptoms of poisoning: nausea, vomiting, abdominal pain, shortness of breath, dizziness, hyperpyrexia, sweating, diarrhea, collapse, convulsions and, consequently, death even a few days after skin contamination. The course of skin poisoning depends on the solvent. When ingested, PCP causes symptoms similar to those of skin poisoning. It causes disorders of metabolism and changes in the circulatory system, liver, and kidneys. After introduction into the body, PCP accumulates in the liver, brain kidneys, spleen, and adipose tissue, which leads to metabolic hyperacidity, enlargement and dysfunction of the liver, weakening of the immune system, increased porphyrins and plasma Δ-aminolaevulinic acid, etc.

In the case of chronic exposure to PCP, pathological changes in the genitals are also found. It accumulates in semen and leads to chromosomal aberrations within peripheral lymphocytes. The toxic effect of PCP is an increase in fetal malformations and their mortality. The smallest lethal dose for humans is PCP delivered to the body via the oral route: 400 mg/kg body weight, i.e., about 28 g, LD 50 inhalation 355 mg/m^3^, LD 50 skin 96 mg/kg. According to the EPA [18,19], pentachlorophenol is a probable human carcinogen in Group B2. As part of the noncancer assessment for PCP (chronic effects—noncancer), EPA determined a Reference Dose (RfD) of 0.03 mg PCP per kg body/day based on rat liver and kidney pathology. In turn, the California Environmental Protection Agency (CalEPA) calculated the reference exposure level for chronic inhalation as 0.1 mg/m^3^. Contaminated drinking water may also be a source of exposure of organisms to PCP. EPA, using mathematical models based on animal studies, calculated the oral unit risk of 3 × 10^−6^ (μgL)^−1^ [18,19].

### 2.2. Lindane

Lindane (Table 1) is one of the hexachlorocyclohexane (HCH) isomers whose names depend on the position of hydrogen atoms in the chemical structure. Only the gamma-HCH (or γ-HCH, commonly called lindane) isomer has insecticidal properties. It has the form of a white, crystalline solid. It is used as an insecticide on fruit, vegetables and forest crops, as well as animals and animal housing. In addition to its insecticidal use, it is also used as a prescription drug (lotion, cream, or shampoo) to treat and/or control scabies (mites) and head lice in humans. Technical purity HCH is a mixture of several isomers, formerly used as an insecticide in the United States, currently not produced in the US [24]. HCH is moderately acutely toxic, and LD50 values for various animal species range from about 60 to over 3700 mg/kg body weight, from 900 to >8000 mg/kg body weight (dermal exposure), and 690 mg/m^3^ (inhalation) [25].

The toxic effects of HCH on animals include neurotoxic symptoms (convulsions, difficulty breathing, and paralysis of the paws), an increase in the relative and absolute weight of organs (liver and kidneys), changes in the liver, kidneys, and testes, and changes in enzyme activity and plasma protein profile, indicative of functional liver injury. Lindane, as a mixture of isomers, does not show mutagenic and genotoxic effects. Single isomers are carcinogenic. Struciński [25] suspects that the α-isomer is the isomer with the highest carcinogenic activity. HCH isomers have an affinity for adipose tissue; they are relatively quickly metabolized and excreted from the body compared to other chloro-organic compounds. Only the β isomer is not rapidly metabolized, which is why it has the ability to bioaccumulate in adipose tissue. HCH metabolism takes place mainly in the liver via oxidases of mixed functions related to cytochrome P-450 [25]. The basic directions of metabolic changes in the liver are dehydrogenation, dehydrochlorination, dechlorination, and hydroxylation [25]. HCH isomers can, among others, disturb neurotransmission, inhibit the activity of sodium-potassium, magnesium, and calcium ATPase, disturb calcium metabolism, disturb the balance of phospholipid cell membranes, and cause oxidative stress in the liver. Moreover, HCH isomers, like other chloro-organic insecticides, by stimulating cytochrome P-450, may modify the metabolism of other compounds in many directions, both endogenous and exogenous, which, especially in the case of chronic exposure, may be one of the most important mechanisms of toxic action [25].

The poisonings reported so far among humans were most often caused by improper use of lindane. The toxic dose is administered as 100–200 mg per kg body weight. The dangerous dose for humans is between 10 and 20 mg/kg and 0.5–2.4 mg/kg when administered orally. Lindane is, therefore, a pesticide of moderate toxicity and does not cause allergies. On the other hand, long-term exposure to lindane is significant and is most often associated with occupational toxicity—chronic inhalation. The value of the so-called Valeur MAK (Threshold limit value—TVL) until 1954 was 0.5 mg/m^3^ for all European countries. For the countries belonging to the Comecon, the established value was 0.1–0.05 mg/m^3^.

### 2.3. Creosote

Creosote (Table 1) was used as a wood preservative from the mid-nineteenth century. Currently, creosote is only used in certified wood preservation plants using high-pressure equipment. According to EPA guidelines, it is recommended to use alternatives to creosote-treated wood due to the toxicity of creosote [5].

Creosote is registered as a preservative to protect wood from fungi, insects, and marine organisms. Its main use as a wood preservative is in the preservation of wood elements on land and in water. It was used to protect the wood with the pressure method of power poles, crossbars and railway sleepers, fences, and fence posts. Foundation beams, beams, sawn timber, and piles were secured with it. Wood impregnated with creosote could only be intended for outdoor use. It is estimated that in the USA, almost all railroad tracks, switches, and bridge beams, as well as about 15% of all power poles, are treated with creosote [5].

Creosote is the name that has been given to a mixture of several oils extracted from tar. According to its composition, there are three types of creosotes: A, B, and C. They all contain over 80% of PAHs, a group of highly toxic compounds. Polycyclic aromatic hydrocarbons (PAHs) constitute a large group of compounds with a ring structure. They dissolve poorly in water. They are formed in the process of pyrolysis of organic substances, as well as incomplete combustion. The creosote mixture consists mainly of acenaphthene, acenaphthylene, naphthalene, phenanthrene, anthracene, fluorene, fluoranthene, chrysene, triphenylene, benzo(a)anthracene, benzo(b)fluoranthene, benzo(k)fluoranthene, benzo(a)pyrene (Table 1) and basic and acid components—creosols, phenols, methylated pyrene derivatives and others [26].

Creosote may contain over 30 different compounds from the group of polycyclic aromatic hydrocarbons (PAHs), the total concentration of which can reach 85% of the product. The PAHs in the creosote mixture are divided into three distinct groups: PAHs with two fused aromatic rings, PAHs with three fused aromatic rings, and PAHs with four and five fused aromatic rings [5].

Compounds from the PAH group have carcinogenic properties, and the presence of creosote causes a cancer risk for consumers due to the presence of benzo(a)pyrene (BaP), according to Commission Dir. 2001/90/EC [27].

Pursuant to the Regulation of the European Parliament (No. 528/2012 of 22 May 2012) [6], creosote used to impregnate railway sleepers was recognized as a non-threshold carcinogen and classified as a carcinogen category 1B, and some of the PAHs were found to be persistent, accumulative and toxic.

The most common routes of poisoning are direct food, inhalation, and skin. Short-term symptoms include eye irritation, nausea, vomiting, and diarrhea. Long-term symptoms include cataracts, kidney and liver damage, and jaundice.

The most important diseases include a high risk of lung cancer and cancer of other internal organs. PAHs also show genotoxic and mutagenic properties. An indicator of air pollution, for example, is the concentration of benzo(a)pyrene. In Poland, since 1995, the value of the maximum allowable concentration (NDS) of this compound was established at the level of 2.0 µg/m^3^; in 1998, the NDS for dibenzo(a, h)anthracene was introduced at the level of 4 µg/m^3^.

In accordance with the provisions of the above directive 2001/09/EC [27], due to the risk of creosote, wood after such treatment cannot be used in the production of construction elements, toys, furniture, garden containers, packaging, etc. It should be mentioned here that human exposure or contact with PAHs may occur not only through contact with creosote, wooden elements and waste containing creosote, but also with the protective agent itself. Food, as well as tobacco smoke, are significant sources of PAHs. Food can be contaminated with PAHs through contact with contaminated elements, but most of all, PAHs are formed as a result of thermal processing such as grilling, baking, and smoking [22].

## 3. Characteristics of Biological Decomposition

Biological degradation consists of the use of substances by microorganisms as a carbon source in metabolic processes (catabolism) and in the processes of cell synthesis (anabolism). Biotransformation of compounds occurs under the action of enzymes. Before being included in the metabolic pathways, macromolecular substances undergo a multistage transformation. The decomposition of compounds such as chloro-organic or polycyclic aromatic hydrocarbons can be carried out with the use of bacteria and fungi capable of metabolizing these compounds [28]. Microorganisms used in the bioaugmentation process should have appropriate metabolomic properties. Recently, research on the possible applications of fungi for bioaugmentation has been widespread. It has been shown that fungi can be biodegradable and used for the biotransformation of lignocellulose biomass. The most important fungi which degrade cellulose and lignin are white rot fungi. Their ligninolytic enzymes, such as manganese peroxidases, lignin peroxidases, and laccases, can break the structure of cellulose and lignin [12]. These enzymes are also efficient in the degradation of many toxic compounds. Compared to bacteria, fungi show features that make bioremediation more effective and less expensive due to: (1) lower sensitivity to the toxic effects of pollutants, (2) ability to survive in conditions of nutrient deficiency, and (3) low sensitivity to environmental factors such as temperature, pH, or humidity.

The above properties of fungi result from their structure and the course of metabolomic processes. Myco-remediation is used primarily in the case of particularly toxic, persistent, and difficult-to-degradable pollutants that cannot be broken down by other microorganisms, as well as compounds with limited solubility in water. The usefulness of myco-remediation is due to the fact that fungi are capable of decomposing many chemical compounds simultaneously. Wood decomposition fungi (soft-rot, white-rot, brown-rot) act on specific components of lignocellulosic substances during decomposition. The brown decay fungi break down the cellulose leaving behind lignin, while the white-rot fungi break down both cellulose and lignin simultaneously. This decomposition leaves the wood brittle and of reduced strength (sponge). Wood decomposition fungi, due to the specific decomposition of components naturally occurring in wood, such as cellulose, lignin, hemicelluloses, and other terpene compounds (essential oils), can be used for the decomposition or biotransformation of other components, including wood impurities with a similar structure containing an aromatic ring.

The initiation of the biodegradation process does not require additional adaptation time necessary to start the synthesis of degradation enzymes. They also do not block enzyme synthesis when the amount of chemical susceptibility is too small. The cultivation of fungi is not expensive as they use lignocellulosic substrates as food substrates. During remediation, fungi cause total or partial mineralization of pollutants; it is also possible to transform trace metals and radioactive elements into poorly soluble forms that are less toxic. So far, *Basidiomycota* fungi have been the most widely used in environmental biotechnology, incl. *Penicillium* sp., *Aspergillus* sp., and various yeast species.

Recently, there has been a growing interest in wood-decaying fungi: *Trametes versicolor*, *Trametes villosa*, *Bjerkandera adusta*, *Lentinula edodes*, and *Pleurotus ostreatus*. It turned out that lignin peroxidase and other fungal enzymes involved in lignin degradation exhibit low substrate specificity and catalyze an equally wide variety of structurally diverse environmental pollutants. Such substances also include those considered resistant to biodegradation, e.g., polycyclic aromatic and aliphatic hydrocarbons, chlorinated organic compounds, pesticides, dyes, or residues of explosives and drugs.

### 3.1. Fungal Degradation of PCP

Until now, the degradation of PCP with the participation of microorganisms has been associated mainlyc with the participation of bacteria. The literature only indicates that fungi are predisposed to the degradation of PCP; data on the metabolism of PCP itself are fragmentary. White decay fungi are among the fungi selected to efficiently metabolize PCP, such as *Amylomyces rouxii*, *Antracophyllum discolour*, *Phanerochaete* sp., *Trametes* sp., *Pleurotus* sp. [13] and basidiomycetes of white and brown rot. Among them, more attention due to better results was given to the genera *Phanerochaete*, *Anthracophyllum*, and *Trametes*. *Zygomycetes*, *Basidiomycetes*, *Ascomycetes*, and *Deuteromycetes* have also been studied for PCP degradation [13,16,29].

**Figure 1:**
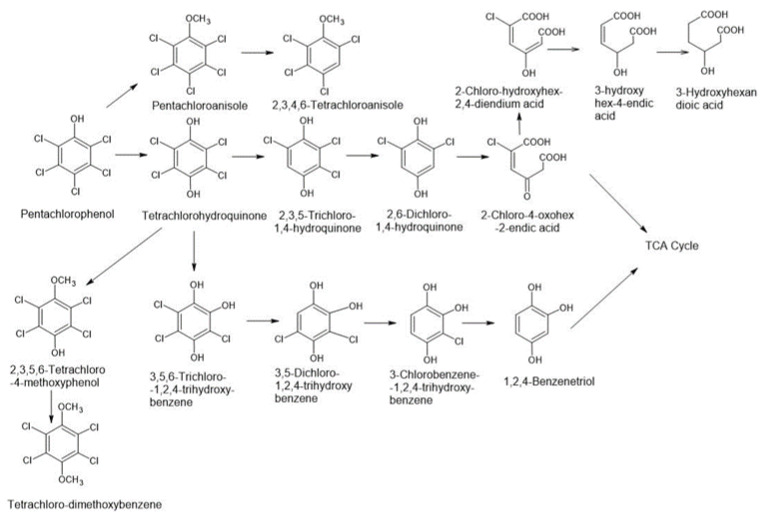
Proposed pathways of pentachlorophenol (PCP) biotransformation using fungi based on literature [31,32].

Based on the available literature, an original metabolic pathway for white rot fungi has been proposed (Figure 1). When bio transformed by these organisms, the conversion of PCP will take place under aerobic conditions. The conversion of PCP under aerobic conditions can take place by exchanging a chlorine atom with a hydroxyl group or with a hydrogen atom. The aromatic ring is broken by introducing oxygen atoms in positions one and two with the simultaneous removal of chlorine, which leads to the formation of chain carboxylic acids and their inclusion in the Krebs cycle [13,17] (Figure 1). The PCP degradation process can also occur by separating chlorine gas atoms, eventually leading to the formation of phenol. Then, the phenol molecule undergoes methylation reactions leading to the formation of toluene derivatives or carboxylation and esterification to flatlands [17]. The obtained intermediates break, and the resulting carbon chains polymerize into octadecanoic acid and hexadecane [17] (Figure 1). Another method of transforming polychlorophenols under aerobic conditions that can lead to the formation of substrates of lower toxicity is the alkylation of the hydroxyl group of chlorophenols, leading to the formation of, among others, chloroanisols, such as pentachloroanisole (PCA) and trichloroanisole (TCA) (Figure 1) [17,30,31]. When PCP is decomposed by white-rot fungi such as *Phanerochaete* [31], PCA is produced as a metabolite (Figure 1). On the other hand, it has been noted that these fungi effectively degrade the substrate PCP, decomposing, depending on the strain used, up to 100% of the initially introduced PCP [13].

White decomposition fungi use ligninolytic enzymes in the described reactions. In contrast, Bosso and Cristinzio [13] believe that the degradation of PCP is not a consequence of enzyme action but rather of co-metabolic reactions. PCP can undergo a hydroxylation reaction by replacing a chlorine atom with a hydroxide, converting PCP into intermediates. The first product of the hydroxylation reaction is tetrachlorohydroquinone (TCHQ), which may be further processed [30,31,32]. By way of dechlorination, it can be converted into 2,3,5-trichloro-1,4-hydroquinone (2,3,5-TCHQ) after another chlorine disconnection—2,6-Dichloro-1,4-hydroquinone (2,6 -DCHQ). The latter (2,6-DCHQ) can transform from the aromatic form after cleavage of the aromatic ring to the aliphatic form as a 2-Chloro-4-oxohex-2-endic acid, which may already be included in the TCA cycle (Figure 1) [13,32]. Tetrachlorohydroquinone (TCHQ) may be further transformed by methylation to 2,3,5,6-Tetrachloro-4-methoxyphenol (TCMP) and further to Tetrachloro-dimethoxybenzene (TCDB). Alternatively, it can be formed after another dechlorination and replacement of the chlorine with the -OH group compound 3,5,6—Trichloro—1,2,4-trihydroxybenzene. This, in turn, may undergo further dechlorination to successively obtain 3,5-dichloro-1,2,4-trihydroxybenzene, 3-chlorobenzene-1,2,4-trihydroxybenzene and 1,2,4-benzenetriol. On the other hand, 1,2,4-benzenetriol, can be included in the TCA cycle.

Another mechanism suggests that fungi use a reaction catalyzed by PH oxidases such as laccases and peroxidases; fungi are able to primarily transform PCP into pentachloroanisole (PCA) [16,29,33]. PCA is a less toxic form of PCP, and because it is a compound with a more lipophilic composition, it passes through the cell membrane and is rapidly bioaccumulated by microorganisms. The last described step in the PCP removal pathway uses the adsorption of PCP to the fungal biomass (live or dead). It was found that some microbial cultures show a special affinity for PCP binding, i.e., adsorption takes place due to charge transfer between PCP and the microbial biomass. Research by McAllister et al. [16] showed that the addition of wood chips contaminated with PCP treated with *Trametes hirsuta* or wood sawdust treated with *Phanerochaete chrysosporium* resulted in more efficient degradation of PCP. Fungi also play an important role in the degradation of PCP-containing waste, including PCP-impregnated wood products. Unlike bacteria, fungi degrade these wastes more easily because of their ability to degrade lignin [16].

Based on the available information, the following fungi were found to be the best candidates for PCP biodegradation in wood waste: *Bjerkandera adusta*, *Trametes versicolor*, *Penicillium* sp. and *Trichoderma* sp. (Table 2).

### 3.2. Fungal Degradation of Lindane

Due to the similar structure, the possible metabolic pathways of lindane show some similarities to the changes taking place during the degradation of PCP by white degradation fungi (Table 3). The basic reaction of the biodegradation of organochlorine compounds is the removal of a halogen atom from a molecule; this process may take place according to three basic mechanisms: (1) hydrolysis—replacement of a halogen atom with a hydroxyl group, (2) reductive dehalogenation—replacement of a halogen atom with a hydrogen atom, and the basic mechanism of anaerobic biodegradation of aliphatic halogen derivatives, (3) dehydrohalogenation—removal of a halogen atom and hydrogen from adjacent carbon atoms with the formation of a double bond.

**Figure 2:**
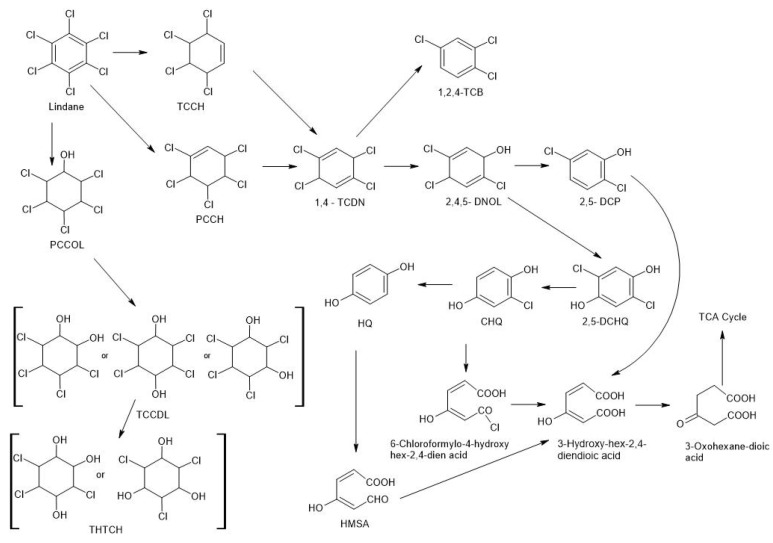
Proposed pathways of lindane biotransformation using fungi based on literature [40,41,42]. Abbreviations: TCCH—3,4,5,6-Tetrachlorocyclohex-1-en; PCCOL—2,3,4,5,6-Pentachlorocyclohexan-1-ol; PCCH—1,3,4,5,6-Pentachlorocyclohex-1-en; 1,4-TCDN—1,3,4,6-Tetrachlorocyclohex-1,4-diene; 2,4,5-DNOL—2,4,5-Trichlorocyclohex-2,5-dien-1-ol; 2,5-DCP—2,5-Dichlorophenol; HQ—Hydroquinone; CHQ—2-Chlorohydroquinone; 2,5-DCHQ—2,5-Dichlorohydroquinone; TCCDL—3,4,5,6-Tetrachlorocyclohex-1,2-diol; 2,3,5,6-Tetrachlorocyclohex-1,4-diol; 2,3,5,6-Tetrachlorocyclohex-1,3-diol; THTCH—3,5,6-Trichlorocyclohex-1,2,4-triol; 2,4,6-Trichlorocyclohex-1,3,5-triol; HMSA—6-Formyl-4-hydroxyhex-2,4-dienic acid; 6-chloroformyl-4-hydroxyhex-2,4-dienic acid; 3-Hydroxy-hex-2,4-diendioic acid; 3-Oxohexane-dioic acid; 1,2,4-TCB—1,2,4-Trichlorobenzene.

During biodegradation under aerobic conditions, a halogen atom is exchanged with a hydroxyl group, and as a result of mineralization, CO_2_, water, and halogen ions are formed (Figure 2). Dehalogenation is a reaction that, in consequence, facilitates further biodegradation and reduces the risk of the formation of toxic intermediates in its further stages. Halogen detachment catalysts are dehalogenases belonging to several classes of enzymes, which are associated with different dehalogenation mechanisms by environmental microorganisms [38]. In the case of lindane, dehalogenation occurs by intramolecular substitution. This process is based on dehydrohalogenation when the hydrogen halide molecule is eliminated with the formation of a double bond (the initial stages of lindane degradation are dehydrohalogenation reactions). In addition to dehalogenation, a key stage in biodegradation is the cleavage of the aromatic ring and, ultimately, the formation of cyclic compounds that can be included in the citric acid cycle.

The central intermediates of the aerobic biodegradation of lindane are benzene-1,2-diol (catechol) derivatives. However, before ring cleavage occurs, catechol or protocatechuic acid is formed. The ring is split due to the catalytic action of enzymes from the dioxygenases group, which allows the attachment of an oxygen molecule. The most common are two different metabolic pathways that differ in the input of ring tearing: ortho type and meta type [39]. Ortho (intradiol) cleavage takes place between adjacent carbon atoms to which the hydroxyl groups are attached. When the starting compound is catechol, a cis, cis-muconic dicarboxylic acid is formed in the presence of catechol 1,2-dioxygenase. The cleavage of the aromatic ring in the protocatechuic acid molecule occurs with the participation of procatechol 3,4-dioxygenase and leads to the formation of 3-carboxy-cis, -cis-muconic acid. In both cases, biodegradation proceeds towards the formation of 3-catechoadipic acid, the degradation products of which are included in the citric acid cycle. Meta (extruded) cleavage takes place between 2 carbon atoms in position α with respect to the diol group. The reaction catalyzed by catechol 2,3-dioxygenase from catechol produces 2-hydroxymucic acid semialdehyde, and in the case of protocatechuic acid cleavage, which occurs in the presence of protocatechol 4,5-dioxygenase, the corresponding mucic acid semialdehyde is also formed. A further reaction leads to the formation of molecules that are then incorporated into the citric acid cycle of the cell.

The literature on the subject indicates that the most effective lindane bio transformants are: *Phlebia* sp., *Trametes versicolor* and *Fusarium* sp.

**Table 3:** Fungi applied for biotransformation of lindane.

Organisms/Lindane Transforming Fungi	Conversion Into Chemical Compounds	Research Object/Lindane Concentration	Bibliography
*Phlebia brevispora* *Phlebia lindtneri*	Several unreported hydroxylation metabolites, including monohydroxylated, dehydroxylated, and trihydroxylated products	Degradation 87.2 and 73.3% of lindane in low nitrogen medium and 75.8 and 64.9% of lindane in high nitrogen medium, respectively.	[43]
*Ganoderma lucidum* GL-2, *Pleurotus ostreatus*, *Fusarium verticilliodes* AT-100, *Rhodotorula* sp. VIT JzN03, *Fusarium poae*, *Fusarium solani*, *Conidiobolous* 03-1-56, *Bjerkandera adusta*, *Cyathus bulleri*	dehydrochlorinase (LinA), dehalogenase (LinB), dehydrogenase (LinC), and reductive dechlorinase (LinD)	Soil, leaves, rotten wood, mean degradation of lindane in %: 59.2–100 in 5–28 days.	[4]
*Ganoderma lucidum* GL-2 strain	dehydrochlorinase (LinA), dehalogenase (LinB), dehydrogenase (LinC)	grown on rice bran substrate for ligninolytic enzyme induction and 40 ppm lindane in liquid as well as solid-state fermentation. The maximum of 75.50% lindane degradation on the 28th day of incubation period, whereas under the solid-state fermentation system, 156.82 U/g laccase, 80.11 U/g manganese peroxidase and 18.61 U/g lignin peroxidase enzyme activities with 37.50% lindane degradation were obtained	[44]
*Trametes versicolor*, *Pleurotus ostreatus*, *Gloeophyllum trabeum*	adsorption onto fungal biomass, organic lindane derivatives	Liquid degradation started between 6 h and 1 day of exposure, and both fungi degraded lindane significantly after 3 days. After 21 days, less than 10% of initial lindane was determined.	[45]
*Trametes versicolor*, *Hypoxylon fragiforme*, *Chondrostereum purpureum*, *Pleurotus ostreatus*, *Gloeophyllum trabeum*	organic lindane derivatives	Amount of lindane was lower in solutions over 70%	[46]
*Fusarium poae* *F. solani*	organic lindane derivatives	Contaminated soil, 0–600 μg mL^−1^ 10th day of incubation degradation by the two fungal strains demonstrated that the biodegradation of lindane by *F. solani* (59.4%) was slightly higher than that by the *F. poae* (56.7%)	[47]

### 3.3. Fungal Degradation of PAHs

**Figure 3:**
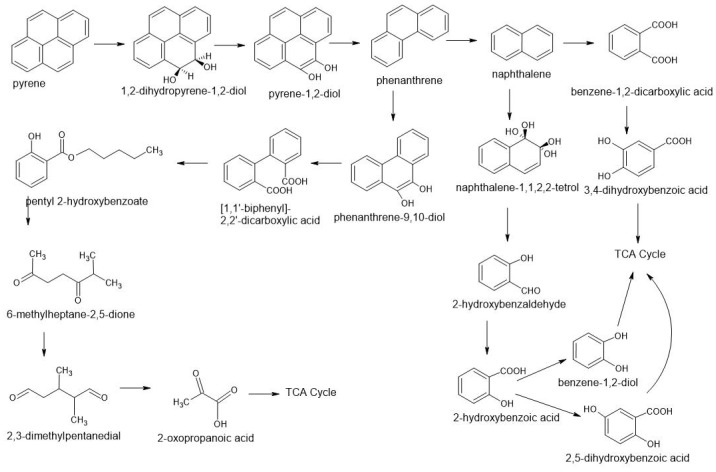
Proposed biotransformation pathways of selected substances from the PAH group using fungi based on literature [14,40,49,50].

Compounds from the group of polycyclic aromatic hydrocarbons that are part of creosote can be decomposed under aerobic and anaerobic conditions; however, the degradation rate of these compounds is clearly higher under aerobic conditions. These hydrocarbons are sensitive to light, oxygen, ozone, and other oxidants. They undergo a photochemical reaction during which diols, quinones, and aldehydes can be formed in the final stage. The reactivity of this group of compounds under aerobic conditions is of great importance during the process of removing these pollutants from the environment [22]. The degradation of these compounds occurs in the environment through substitution reactions and attachment reactions in which unsaturated bonds are destroyed. The most effective organisms in decomposing pollutants from this group include bacteria, fungi, actinomycetes, cyanobacteria, and algae, as well as mixed populations of bacteria or fungi [28]. PAHs decomposing fungi include, among others: *Cunnighamella elegant*, *Rhizoctonia solani*, *Trametes versicolor*, *Chrysosporium lignorum*, *Aspergillus terreus*, *Aspergillus flavus*, *Penicilium trodum* (Table 4). These fungi can break down compounds from the PAHs group as single components of the inoculum and in the form of mixed mushroom cultures [48]. It has been proven that white decomposition fungi such as *Nematoloma frowardii* produce enzymes that are involved in the oxidation of mono-aromatic and poly-aromatic compounds [28]. The degradation process of polycyclic aromatic hydrocarbons (PAHs) can give rise to many intermediate degradation products. Initially polycyclic, successively mono-ring for complete mineralization and obtaining CO_2_ and H_2_O. The proposed metabolic pathways in the first step include the oxidation of PAHs and the inclusion of two oxygen atoms (Figure 3). The compounds are then converted to dihydroxy forms. After hydroxylation of the first benzoic ring, cleavage and conversion to pyruvic acid and carbon dioxide take place. Then another ring disintegrates [28]. The general scheme of PAH degradation is based on two steps: first, the substituent is degraded, and next, the aromatic ring is cleaved. The first phase of enzymatic transformations of PAHs leads to the formation of chlorocatechin derivatives. Subsequent transformations lead to the production of 1,2-diphenol derivatives. Then there is cleavage leading to the rupture of the benzene ring and further transformation into compounds included in the Krebs cycle, gluconeogenesis or ß-oxidation [28].

These reactions take place as a result of the action of ligninolytic enzymes capable of the biodegradation of lignin polymers. The group of enzymes includes lignin peroxidase, manganese peroxidase, and laccase. Lignin peroxidase (LiP) is one of the most important enzymes biosynthesized by White-Rot Fungi. It is characterized by low substrate specificity, which means that it can bind to many substances or their analogs. The action of the enzyme is based on the presence of H_2_O_2_, the reduction of which leads to the simultaneous oxidation of organic substrates. Lignin peroxidase molecules are hemoproteins; they contain heme, which is the active center of the enzyme. They also constitute a protective barrier for cells against oxidative stress. LiP causes, among others, aromatic ring opening, dimethylation, phenol dimerization, and cleavage of the C-C bond by generating free radicals. The mechanism of action of LiP is based on the fact that the heme group under the influence of H_2_O_2_ is oxidized and forms a complex with the degraded compound, leading to the formation of free radicals in their forms, which results in the formation of a complex. The fungus activity was confirmed by the decrease in the concentration of the tested PAH compounds during the experiments. Only a few publications state into which metabolites these compounds can be broken down. Andersson et al. (2003) [40], during decomposition with the use of *Pleorutus ostreatus* and *Anodia vaillantii*, observed in studies of artificially contaminated soil the transformation of the given compounds fluorene, phenanthrene, pyrene, benz[a]anthracene into compounds such as 9-fluorenone, benz[a]anthracene-7.12-dione, 4-hydroxy-9-fluorenone, 4-oxapyrene-5-one. Using also white rot fungus *Pleurotus ostreatus* D1 and the litter-decomposing fungus *Agaricus bisporus* F-8 [49], the decomposition of two isomeric three-ringed polycyclic aromatic hydrocarbons (phenanthrene, anthracene) into Quinone was observed and further decomposition to other metabolites such as phthalic acid.

The strains of fungi used, matrices used during decomposition, and the duration of the experiment were taken into account (Table 4).

The conducted literature studies are summarized in Table 5, where the possibilities of fungal organisms to simultaneously deactivate toxic substances such as PCP, lindane, and PAHs are indicated. The collected and systematized information became the basis for carrying out research on the preparation of a biological preparation for the simultaneous transformation and detoxification of wood raw materials contaminated with the above-mentioned toxic substances.

## 4. Conclusions and Perspectives

Biological degradation by fungi is one of the ways of eliminating undesirable compounds from waste materials before they are incorporated into the environment. Biodegradation of such compounds also increases the possibility of using such waste in the perspective of a circular economy. In this review, we aimed to summarize the possibility of using white rot fungi for the biotransformation of wood preservatives such as PCP, lindane, and PAHs. Scientific research on the topic indicates the possibility of using organisms to degrade individual compounds or to create a combination or consortium of fungi decomposing these specific compounds at the same time. Based on the literature results, the most appropriate fungi able to biodegrade the hazardous compounds applied as wood preservatives are *Trametes versicolor*, *Bjerkandera adusta*, and *Phanerochaete chrysosporium*. The use of these fungi led to a significant reduction in the concentration of PCP, lindane, and PAHs, which means that the composts produced with the application of these fungi in inoculum can be used as components of substrates for plant cultivation.

Future research should be completed to confirm the reduced ecotoxicity and phytotoxicity of the wood waste treated by fungi and also to optimize the consortium of fungi, which can be applied for the bioremediation process in order to achieve higher efficiency of hazardous compound removal. In summary, this paper constitutes a compendium of knowledge presenting additional aspects of waste valorization and could be introduced into practice in many different areas, including forestry, agriculture, and horticulture.

## Figures and Tables

**Table 1:** Characteristics of selected wood protection compounds (PCP, lindane, creosote).

Compound (Cas Number and Names)	Occurrence/Application	Hazard/Toxicity	Bibliography
Pentachlorophenol CAS #: 87-86-5	pesticide herbicide, insecticide, fungicide, algicide, disinfectant component of anti-fouling paints	da Non-flammable substance. Practically insoluble in water Chemical formula: C6Cl5OH Structural formula: 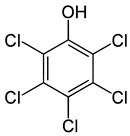 Molecular weight: 266.4 It decomposes at 309 °C Melting point: 191 °C Density: 1.98 g/cm^3^ Water solubility, g/100 mL at 20 °C: 0.001 Vapor pressure, Pa at 20 °C: 0.02 Relative vapor density (air = 1): 9.2 Relative density of the vapor/air-mixture at 20 °C (air = 1): 1.00 Octanol/water partition coefficient as log Pow: 5.01. Hygienic Standards: TLV: 0.5 mg/m^3^, as TWA. TLVSTEL1 1 mg/m^3^. (skin); A3 (agents proven to be carcinogenic in animals and not known to be carcinogenic in humans); DSB. MAK: skin absorption (H); carcinogen category: 2.	[16,18,19,20]
Lindane (γ-hexachlorophenol) Hexachlorocyclohexane 1,2,3,4,5,6-Hexachloro-cyclohexane gamma-1,2,3,4,5,6-Heksachlorocykloheksan gamma-BHC gamma-HCH CAS #: 58-89-9	Pesticide Insecticide (on fruits, vegetables, forest crops, animals, and on animal premises)	Non-flammable substance. Practically insoluble in water. Chemical formula: C_6_H_6_Cl_6_ Structural formula: 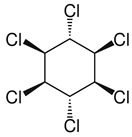 Molecular weight: 290.8 Boiling point: 323 °C Melting point: 113 °C Density: 1.9 g/cm^3^ Solubility in water, g/100 mL at 20 °C: 0.0007 (very poor) Vapor pressure, Pa at 20 °C: 0.0012 Relative density of the vapor/air-mixture at 20 °C (air = 1): 1 Octanol/water partition coefficient as log Pow: 3.61–3.72 Hygienic standards TLV: 0.5 mg/m^3^, as TWA. TLVSTEL1 1 mg/m^3^. (skin); A3 (agents proven to be carcinogenic in animals and not known to be carcinogenic in humans); DSB. MAK: skin absorption (H); carcinogen category: 2	[4,21]
Creosote oil Coal creosote Example of CAS # acenaphthene: 83-32-9; acenaphthylene: 208-96-8; anthracene:120-12-7; benzo[a]pyrene: 50-32-8	Pesticide, Fungicide, Insecticide, Miticide, Sporicide Products for wood used outdoors, e.g., railroad ties and utility poles, crossarms, fences, fence posts, foundation timbers, timbers, lumber, and pilings. Treated wood intended for exterior/outdoor uses only.	Black to brown, oily liquid with a characteristic odor. The formulas of the most important PAHs(Examples): Naphthalene 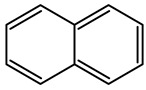 Anthracene 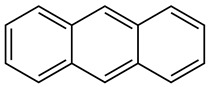 Pyrene 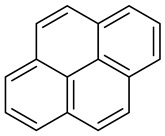 Benzo(a)pyrene 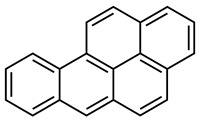 Boiling point: 200–400 °C Melting point: ~20 °C Density: 1.0–1.17 g/cm^3^ Water solubility: very poor Vapor pressure, kPa at 20 °C: ~6 Flash point: >66 °C c.c. Auto-ignition temperature: 335 °C Hygienic Standards ILO and WHO 2018: not given	[22,23]

**Table 2:** Fungi applied for biotransformation of pentachlorophenol (PCP).

Organisms/PCP Transforming Fungi	Conversion Into Chemical Compounds	Research Object/PCP Concentration	Bibliography
*Anthracophyllum discolor*	PCA; TCHQ	Soil 28 days 100–300 mg/kg	[13]
*Agrocybe perfecta* CCB161	PCA, Chloride ions	Soil 90 days 1180–1278 mg/kg	[13]
*Armillaria gallica* 1057	2-methyl-1,3 benzenediol; 6-phenyl-dodecane	Liquid 7 days 25 mg mg/L	[13]
*Armillaria mellea* M51	2-methyl-1,3 benzenediol	Liquid 7 days 25 mg/L	[13]
*Bjerkandera adusta* ATCC 62023	PCA; 2,3,4,6-tetrachloroanisole	Soil 4 weeks 100 µg/g	[13]
*Bjerkandera adusta* ATTC 90940	PCA; TCHQ	Soil 28 days 100–300 mg/kg	[13]
*Chrysonilia sitophila* DSM 16514, *Trichoderma longibrachiatum*, *Mucor plumbeus*, *Penicillium janczewskii* *P. glandicola*	CHQ	Liquid 50–60 days 1–20 mg/L	[13,34]
*Ganoderma lucidum* HK-1	2-methyl-1,3 benzenediol; 1-octyl-benzene	Liquid 7 days 25 mg/L	[13]
*Irpex lacteus* ATCC 11245	PCA; 2,3,4,6-tetrachloroanisole	Soil 4 weeks 100 µg/g	[13]
*Lentinula edodes* LE2	PCA, 2,3,4,6-tetrachloroanisole (TCA), Tetrachlorophenol	Soil 10 weeks 200 mg/kg	[13,33]
*Lentinula edodes* L68	1-chloro-3-methoxy-benzene	Liquid 7 days 25 mg/L	[13]
*Mucor ramonissimus* IM 6203 6203	2,3,5,6-TCHQ; pentachloromethoxybenzene	Liquid 240 h 10 mg/L	[13,17]
*Mucor plumbeus* DSM16513 16513	TriCHQ; TCHQ	Liquid 4 days 15–18.8 µM	[13,35]
*Mucor ramosissimus* IM 6203	2,3,5,6-TCHQ	Liquid 7 days 10 mg/L	[13,17]
*Penicillium adametzii*	TeCBQ	Liquid 50–60 days 1–20 mg/L	[13,34]
*Penicillium* *corylophilum*	CHQ	Liquid 50–60 days 1–20 mg/L	[13,34]
*Penicillium decumbens*	DCBQ	Liquid 50–60 days 1–20 mg/L	[13,34]
*Penicillium glabrum* DSM 16516	CHQ	Liquid 50–60 days 1–20 mg/L	[13,34]
*Penicillium glandicola*	CHQ	Liquid 50–60 days 1–20 mg/L	[13,34]
*Penicillium janczewskii*	CHQ	Liquid 50–60 days 1–20 mg/L	[13,34]
*Penicillium variabile*	CHQ	Liquid 50–60 days 1–20 mg/L	[13,34]
*Peniophora cinerea* CCB204	PCA; Chloride ions	Soil 90 days 1180–1278 mg/kg	[13]
*Phanerochaete chrysosporium* ATCC 42725	PCA; 2,3,4,6-tetrachloroanisole	Soil 4 weeks 100 µg/kg	[13]
*Phanerochaete* *chrysosporium* BMK-F-1767	Chloride ions	Liquid 3 days 250 mg/L	[13]
*Phanerochaete* *chrysosporium* M1	3,3-dimethyl-cyclohexanol	Liquid 7 days 25 mg/L	[13]
*Phanerochaete sordida* HHB-8922-Sp	PCA; 2,3,4,6-tetrachloroanisole	Soil 4 weeks 100 µg/kg	[13]
*Phanerochaete sordida*	PCA	Soil 56 days 175 ppm	[13]
*Phlebia acanthocystis* TMIC34875 *Phlebia tremellosa* TMIC30511 *Phlebia aurea* TMIC33908	pentachloroanisole (PCA) p-tetrachlorohydroquinone (TCHQ) TCHQ transformed into TCMP and TCDB	Liquid 10 days	[31]
*Pleurotus pulmonarius*	TCHQ; TCP	Liquid 2 days 2–100 ppm	[13]
*Polyporus* sp. Cv-1	1-chloro-3-methoxy-benzene	Liquid 7 days 25 mg/L	[13]
*Psilocybe castanella* CCB444	Chloride ions	Soil 90 days 1180–1278 mg/kg	[13]
*Trametes versicolor* HR131	PCA	Soil 1–2 years 1000 mg/kg	[13]
*Trametes versicolor* MD-277	PCA; 2,3,4,6-tetrachloroanisole	Soil 4 weeks 100 µg/kg	[13]
*Trametes versicolor* PRL 572	PCA; 2,3,4,6-tetrachloroanisole	Soil 42 days 996 µg/g	[13,36]
*Trametes villosa* CCB176	Chloride ions	Soil 90 days 1180–1278 mg/kg	[13]
*Trametes villosa* CCB213	PCA; Chloride ions	Soil 90 days 1180–1278 mg/kg	[13]
*Trichoderma harzianum* CBMAI 1677	pentachloroanisole (PCA) 2,3,4,6-tetrachloroanisole (2,3,4,6-TeCA)	Liquid 7 days 10–50 mg/L	[30]
*Trichoderma longibrachiatum* DSM 16517	DCBQ	Liquid 50–60 days 1–20 mg/L	[13]
*Phlebia acanthocystis*	pentachloroanisole and p-tetrachlorohydroquinone, tetrachloro-4-methoxyphenol, tetrachloro-1,4-dimethoxybenzene, p-tetrachlorohydroquinone	potato dextrose agar medium 10 days of incubation remove 100% and 76% of PCP (25 μM) in low-nitrogen and potato dextrose broth media, respectively	[31]
*Aspergillus sydowii* DL6A; *Apergillus versicolor* DL5A; *Cladosporium oxysporum* DL5G; *Fusarium proliferatum* DL11A; *Trichoderma harzianum* CBMAI 1677	pentachloroanisole (PCA); 2,3,4,6-tetrachloroanisole (2,3,4,6-TeCA)	Solid culture medium (3% malt), initial concentration of 20 mgL^−1^ of PCP) using a validated method.	[30]
*Trametes* sp. *Phanerochaete* sp., *Anthracophyllum* sp., *Armillaria* sp., *Bjerkandera* sp., *Ganoderma* sp., *Lentinula* sp., *Penicillium* sp, *Trichoderma* sp., *Rhizopus* sp. *Plerotus* sp.	p-tetrachlorohydroquinone, tetrachloro-4-methoxyphenol, tetrachloro-1,4-dimethoxybenzene, 2,3,4,6-tetrachloroanisole (2,3,4,6-TeCA), non-chlorinated or chlorinated phenol derivatives	Soils, starting concentration (μM): 54.2–100.00; PCP degradation yield (%): 33–76.	[13]
*Cunninghamella* sp. UMAS SD12	-	Liquid, preliminary PCP biodegradation trial performed in minimal liquid medium supplemented with 20 mg/L of PCP, the degradation up to 51.7% of PCP in 15 days.	[29]
*Mucor plumbeus*	Tetrachlorohydroquinone and several phase II conjugates	Soil, degradation up to 60% of PCP in 20 days od incubation.	[37]
*Mucor plumbeus*	glucose, sulfate and ribose conjugates, and identified for the first time in fungi sulfate–glucose conjugates, tetra- and tri-chlorohydroquinones, sulfate–glucose conjugates	Liquid environmental pollution, percentage of PCP biodegradation ranged from 29% to 69%.	[35]

**Table 4:** Fungi applied for biotransformation of PAHs.

Organisms/Pahs Transforming Fungi	Conversion Into Chemical Compounds	Research Object/ Pahs Concentration	Bibliography
*Pleurotus ostreatus* *Antrodia vaillantii*	9-fluorenone, benz[a]anthracene-7,12-dione, 4-hydroxy-9-fluorenone 4-oxapyrene-5-one	Soil artificially contaminated 12 weeks	[40]
*Phanerochaete velutina*	Full degradation	starting concentration 3500 mg·kg^−1^ sum of 16 PAH laboratory scale: starting concentration 3500 mg kg^−1^, sum of 16 PAH 96% of 4-ring PAHs and 39% of 5- and 6-ring PAHs were removed in three months In the uninoculated microcosms, 55% of 4-ring PAHs and only 7% of 5- and 6-ring PAHs were degraded.	[42]
*Bjerkandera adusta* (Willd.: Fr.)Karst.; *Gymnopilus sapineus* (Fr.)Mre.; *Hypholoma fasciculare* (Huds.: Fr.)Kumm.; *Kuehneromyces mutabilis* (Schaeff.: Fr.)Sing. & Smith; *Lenzites betulina* (L.: Fr.)Fr.; Pleurotus sp. (Argentina); *Pleurotus ostreatus* (Jacq.: Fr.)Kumm.	phenanthrene (PHEN, 3 R), anthracene (ANTH, 3 R), fluoranthene (FLUA, 4 R), pyrene (PYR, 4 R), perylene (PER, 5 R), benzo[g,h,i]perylene (BENZ, 6 R), and coronene (COR, 7 R)	Three to 12 days after spiking, 22 to 38% of the PAH could no longer be recovered from the soils. At 287 days, 88.5 to 92.7%, 83.4 to 87.4%, and 22.0 to 42.1% of the 3-, 4-, and 5- to 7-R PAH, respectively, had disappeared from the unsterile, uninoculated soils.	[41]
*Phanerochaete chrysosporium* IMI 232175 *Pleurotus ostreatus* IMI 341687, *Coriolus versicolor* IMI 210866 Wye isolate #7	organic derivatives nontoxic	Wheat straw and non-sterile coal-tar contaminated soil to determine their potential to degrade polycyclic aromatic hydrocarbons (PAHs)	[51]
*Podoscypha elegans* FTG4	phenanthrene (PHE) and pyrene (PYR)	- in-vitro and in-vivo conditions 99% of PHE and 98.9% of PYR from the degradation medium (20 mg L^−1^ concentration individually), although in the in-vivo condition, it reached up to 50.6% of PHE and 48% of PYR (50 mg kg^−1^ concentration individually).	[14]
*Pleurotus ostreatus* D1, *Agaricus bisporus* F-8	two isomeric three-ringed polycyclic aromatic hydrocarbons, phenanthrene and anthracene	liquid medium, mean degradation 48%	[49]
*Scopulariopsis brevicaulis*	phenanthrene, fluoranthene, Pyrene, benzo[a]pyrene	liquid medium phenanthrene (60% removed), fluoranthene (62%), pyrene (64%), benzo[a]pyrene (82%) 30 days of incubation; a PAH-contaminated soil removed 77% of total PAHs from the soil with the addition of the PZ-4 suspension, phenanthrene (89% removed) and benzo[a]pyrene (75%) Incubation for 28 days	[50]

**Table 5:** Fungal strains and recorded biotransforming activity of selected organic substances (PCP, Lindane, PAHs) on the basis of the literature studies. “+” Fungi effective in the biotransformation of selected compounds (PCP, Lindane, PAHs).

Selected Fungi Organism	PCP	Lindane	PAHs
*Aspergillus terreus*			+
*Aspergillus flavus*			+
*Bjerkandera adusta*	+	+	+
*Chrysosporium lignorum*			+
*Cunninghamella elegans*			+
*Fusarium solani*		+	+
*Ganoderma lucidum*	+	+	
*Irpex lacteus*	+		+
*Penicilium trodum*			+
*Phanerochaete chrysosporium*,	+	+	+
*Pleurotus ostreatus*		+	+
*Podoscypha elegans*			+
*Rhizoctonia solani*,			+
*Trametes versicolor*	+	+	+
*Scerotium rolfsii*			+

## Data Availability

Data sharing is not applicable to this article.

## Sample Availability

Not available.
